# *Pichia pastoris*-Expressed Dengue 2 Envelope Forms Virus-Like Particles without Pre-Membrane Protein and Induces High Titer Neutralizing Antibodies

**DOI:** 10.1371/journal.pone.0064595

**Published:** 2013-05-23

**Authors:** Shailendra Mani, Lav Tripathi, Rajendra Raut, Poornima Tyagi, Upasana Arora, Tarani Barman, Ruchi Sood, Alka Galav, Wahala Wahala, Aravinda de Silva, Sathyamangalam Swaminathan, Navin Khanna

**Affiliations:** 1 Recombinant Gene Products Group, International Centre for Genetic Engineering and Biotechnology, New Delhi, India; 2 Department of Infectious Diseases, Ranbaxy Research Laboratories, Udyog Vihar, Gurgaon, Haryana, India; 3 Department of Microbiology and Immunology, University of North Carolina School of Medicine, Chapel Hill, North Carolina, United States of America; University of Pittsburgh, United States of America

## Abstract

Dengue is a mosquito-borne viral disease with a global prevalence. It is caused by four closely-related dengue viruses (DENVs 1–4). A dengue vaccine that can protect against all four viruses is an unmet public health need. Live attenuated vaccine development efforts have encountered unexpected interactions between the vaccine viruses, raising safety concerns. This has emphasized the need to explore non-replicating dengue vaccine options. Virus-like particles (VLPs) which can elicit robust immunity in the absence of infection offer potential promise for the development of non-replicating dengue vaccine alternatives. We have used the methylotrophic yeast *Pichia pastoris* to develop DENV envelope (E) protein-based VLPs. We designed a synthetic codon-optimized gene, encoding the N-terminal 395 amino acid residues of the DENV-2 E protein. It also included 5’ pre-membrane-derived signal peptide-encoding sequences to ensure proper translational processing, and 3’ 6× His tag-encoding sequences to facilitate purification of the expressed protein. This gene was integrated into the genome of *P. pastoris* host and expressed under the alcohol oxidase 1 promoter by methanol induction. Recombinant DENV-2 protein, which was present in the insoluble membrane fraction, was extracted and purified using Ni^2+^-affinity chromatography under denaturing conditions. Amino terminal sequencing and detection of glycosylation indicated that DENV-2 E had undergone proper post-translational processing. Electron microscopy revealed the presence of discrete VLPs in the purified protein preparation after dialysis. The E protein present in these VLPs was recognized by two different conformation-sensitive monoclonal antibodies. Low doses of DENV-2 E VLPs formulated in alum were immunogenic in inbred and outbred mice eliciting virus neutralizing titers >1∶1200 in flow cytometry based assays and protected AG129 mice against lethal challenge (*p*<0.05). The formation of immunogenic DENV-2 E VLPs in the absence of pre-membrane protein highlights the potential of *P. pastoris* in developing non-replicating, safe, efficacious and affordable dengue vaccine.

## Author Summary

Dengue, a viral disease spread to humans by mosquitoes, is endemic to more than a hundred countries. There are four closely related dengue viruses (DENVs) that cause this disease and a preventive vaccine to protect against all four is actively sought. Unexpected hurdles, in weakened virus vaccine development which revealed potential safety risk issues, has spurred renewed interest in non-viral dengue vaccines. Infectious genetic material-free virus-like particles (VLPs), composed only of the viral coat proteins can induce robust immunity without causing infection.

Using recombinant DNA technology, we have created non-infectious DENV VLPs made of only the major DENV envelope protein important for eliciting virus-specific immunity, but lacking the pre-membrane protein implicated in induction of disease-enhancing antibodies. These VLPs elicit very high levels of virus-neutralizing antibodies which protected mice significantly against lethal DENV challenge. The encouraging data obtained for VLPs specific to one of the four DENVs warrant the development of VLPs specific to the remaining three. The use of the high yielding yeast system for producing these VLPs holds great promise for the development of dengue vaccine that may be not only safe and efficacious but also inexpensive, for use in the resource-poor nations where dengue is endemic.

## Introduction

Dengue is an arboviral disease, which threatens almost half the global population, and has emerged as the most significant of current global public health challenges [1], [2]. It is spread to humans by mosquitoes, and is caused by four closely related, but antigenically distinct, serotypes of dengue viruses (DENV-1, -2, -3 and -4), all of which belong to the genus *Flavivirus*, of the family *Flaviviridae* [3]. Dengue is prevalent in over a hundred, mostly economically resource-poor, tropical and sub-tropical countries that collectively represent >2.5 billion people. According to the World Health Organization, there are ∼50 million DENV infections each year globally, of which ∼500,000 result in severe disease, claiming ∼12,500 lives [4]. A prophylactic dengue vaccine has remained elusive, mainly because of the complex pathology of the disease which mandates a successful vaccine to confer simultaneous immunity against all four DENV serotypes.

DENV infections may be asymptomatic or manifest a spectrum of clinical disease ranging from mild, self-limiting dengue fever to severe and potentially fatal dengue hemorrhagic fever (DHF) and dengue shock syndrome (DSS). Aside from high fever, DHF/DSS is characterized by very high levels of viremia, thrombocytopenia, abnormal hemostasis and vascular leakage due to endothelial damage and in the absence of medical attention can result in very high case fatality rates [1], [3], [4]. While infection with any DENV serotype can provide durable long-lasting homotypic immunity [5], it tends to predispose patients to severe dengue disease upon subsequent infection with a heterotypic DENV [6]. Cross-reactive antibodies from a prior infection are implicated in facilitating increased uptake of heterotypic DENVs by Fc receptor-bearing monocytes and macrophages through antibody-dependent enhancement (ADE) leading to higher viral load and severe disease [3], [7]. As partial immunity can potentially sensitize a vaccinee to severe disease, a safe dengue vaccine must be tetravalent, capable of providing durable immunity simultaneously to all four DENV serotypes. Multiple approaches are being pursued towards developing a safe and efficacious dengue vaccine. These include live attenuated vaccines (LAVs), purified inactivated virus vaccines, plasmid- and viral vector-based vaccines, and recombinant subunit vaccines [2], [8]–[10]. Of these, the LAVs are in advanced stages of development with one of these, the chimeric yellow fever vaccine vector-based dengue vaccine (CYD), currently in phase III trials. However, LAVs pose unique challenges arising from replication interference between the monovalent LAVs in tetravalent formulations, resulting in reduced neutralizing antibody titers to some DENV serotypes [8], [11], [12]. As a result, there is now renewed interest in non-replicating dengue vaccines [8].

One approach to non-replicating dengue vaccines may be based on virus-like particles (VLPs). Many viral coat proteins [13], including those of DENV [14]–[17], when expressed in heterologous hosts, manifest an intrinsic propensity to self-assemble into VLPs that are similar to the parent virions. These VLPs have unique advantages as vaccine antigens. Their architecture that presents a high density of repetitive epitope arrays make them highly immunogenic. In addition, their lack of infectious viral genome makes them safe [18], [19]. Thus, these VLPs offer the means to trigger a robust immune response in the absence of any infection. This has been borne out by the success of VLP vaccines against hepatitis B and human papilloma virus infections [13].

For DENV, it has been shown that co-expression of two structural proteins, the pre-membrane (prM) protein and the envelope (E) glycoprotein, can result in the generation of discrete VLPs in yeast [14], [15], insect [16] and mammalian [17] expression hosts. Of these two DENV structural antigens, the E glycoprotein is the key vaccine immunogen. It is a large protein of about 500 amino acid (aa) residues, organized into three discrete domains and held together by six S-S bridges [20], and constitutes the major structural component of the virion surface [21]. It is a multifunctional protein involved in host receptor recognition and host membrane fusion during infection of susceptible cells, and the major target of virus-neutralizing antibodies elicited during a natural infection [3], [22]. On the other hand, the prM protein which has a role in virus maturation [22] has been implicated in the elicitation of antibodies that can mediate ADE [23], [24]. We used the N-terminal 80% of the DENV-2 E molecule (also known as the ectodomain), as reports in the literature have indicated that deletion of the C-terminal hydrophobic membrane anchor stem region enhances its immunogenicity [25]. We expressed recombinant DENV-2 E ectodomain, lacking the C-terminal 100 aa residues of full length E (referred to as DENV-2 E, henceforth for simplicity) using the methylotrophic yeast *Pichia pastoris*. We have used this yeast to express other S-S linked native [26] and chimeric [27] viral antigens that manifest self-assembly into VLPs. Importantly, from the perspective of inexpensive vaccine development, especially for use in resource-poor countries, it offers several advantages including the availability of a very strong methanol-inducible alcohol oxidase 1 (*AOX1*) promoter, cultivation to very high cell density in simple inexpensive media, the capacity for high productivity and the ability to perform post-translational modifications [28]. Surprisingly, despite the many advantages offered by *P. pastoris*, it has not been exploited effectively so far for the production of DENV E antigens for use as sub-unit vaccines (Table S1).

In this work, we have sought to explore the utility of *P. pastoris* as an expression host for developing dengue sub-unit vaccines. Specifically, we have addressed the following questions: Can DENV-2 E be expressed efficiently in this yeast? Would it self-assemble into VLPs in the absence of prM? If it did, would such VLPs be useful as potential subunit vaccines? We report for the first time that DENV-2 E protein indeed assembles into discrete VLPs without prM. We further present data demonstrating the immunogenicity and protective efficacy of these DENV-2 E VLPs using small animal models.

## Methods

### Ethics statement

Animal experiments were performed in accordance with National animal ethics guidelines of the Government of India after approval by Institutional Animal Ethics Committees of International Centre for Genetic Engineering & Biotechnology, New Delhi, Ranbaxy Research Laboratories, Gurgaon, and Abexome Biosciences, Bangalore.

### *DENV-2 E* gene, cells, viruses, antibodies and other reagents

The *DENV-2 E* gene (∼1.4 Kb, GenBank accession no: JX292265), codon-optimized for *P. pastoris* expression, was obtained from GenScript (New Jersey, USA). *P. pastoris* expression host (strain KM71H) and the integrative plasmid pPICZ-A were purchased from Invitrogen Life Technologies (Carlsbad, USA). The plasmid provides the methanol-inducible *AOX1* promoter for heterologous gene expression. The viruses DENV-1, DENV-2, DENV-3 and DENV-4 have been described before [29]. Cell lines Vero, BHK 21 and C6/36 were from American Type Culture Collection (ATCC), Virginia, USA. The U937 cell line expressing dendritic cell-specific intercellular adhesion molecule 3-grabbing non-integrin (DC-SIGN) has been reported before [30]. Ni NTA Super-flow resin, Ni-NTA His-Sorb plates and anti-His monoclonal antibody (mAb, 34660) were purchased from Qiagen (Hilden, Germany). DENV-2 EDIII-specific mAb 24A12 was generated in-house [31]. Pan-DENV prM-specific 2H2 mAb has been reported earlier [32]. Pan-DENV E-specific 4G2 mAb was from ATCC. Anti-mouse IgG antibody-horseradish peroxidase (HRPO) and -fluorescene isothiocyanate (FITC) conjugates were from Calbiochem, La Jolla, CA. Concanavalin A (Con A) –HRPO conjugate, the HRPO substrate 3, 3′, 5, 5′-Tetramethylbenzidine and acid-washed glass beads (425–600 microns) were from Sigma-Aldrich, St. Louis, MO. Alexa Fluor 488 for labeling mAbs was from Life Technologies (Molecular Probes, Inc). Uranyl acetate was from TAAB Laboratories Equipment Ltd (UK). BCA protein assay reagent was from Thermo Scientific, Rockford, USA.

### Generation of DENV-2 E-expressing *P. pastoris* clone

The synthetic *DENV-2 E* gene was cloned under the control of *AOX1* promoter of pPICZ-A vector and integrated into the host genome of *P. pastoris* strain KM71H. Transformants obtained through zeocin selection were screened for *DENV-2 E* gene integration [33] and its expression [34], as described before.

### Induction of expression and preparation of extracts

Yeast cultures grown to logarithmic phase in buffered glycerol-containing medium (BMGY) were induced in 1% methanol-containing medium (BMMY) for 72 hours. Induced cells (∼100 OD_600_) were lysed using glass beads and separated into supernatant (S) fraction and pellet (P). The latter was extracted in 6 M guanidine-HCl (GuHCl). The S and P fractions were analyzed for the presence of the recombinant DENV-2 E antigen by Western blot assay. Relative levels of recombinant DENV-2 E protein were assessed by His-Sorb ELISA using mAb 24A12 [34].

### Ni2+-affinity purification of recombinant DENV-2 E protein

Induced cell pellet obtained from 3L starting culture was washed in sterile 1× phosphate buffered saline (PBS), re-suspended in 300 ml cell suspension buffer, CSB (50 mM Tris-HCl (pH 8.5)/500 mM NaCl), and lysed with glass beads in a Dynomill. The lysate was spun down to separate out the membrane-enriched P fraction and stirred in membrane extraction buffer MEB (CSB supplemented with 6 M GuHCl and 30 mM imidazole) for ∼4 hours at room temperature. The resultant extract was clarified by centrifugation (13,000 rpm, 1 hour, 4°C) and filtration (0.45 µ), and bound to Ni^2+^-NTA resin (25 ml of a 50% slurry, pre-equilibrated in MEB) in batch mode and packed into a chromatographic column connected to an AKTA purifier. The column was washed extensively with MEB to replace 6 M GuHCl with 8 M urea, and eluted using a step imidazole gradient in 8 M urea-MEB. Column fractions containing purified protein, based on SDS-PAGE analysis, were pooled and dialyzed against 20 mM Tris-HCl (pH 8.5) buffer containing 50 mM NaCl.

### Characterization of recombinant DENV-2 E protein

Recombinant DENV-2 E protein in crude extracts (S and P fractions), chromatographic column fractions and purified protein preps was analyzed by SDS-PAGE and Western blotting, using either mAb 24A12 or penta-His mAb, in conjunction with anti-mouse IgG-HRPO conjugate. The purified DENV-2 E protein was also analyzed in ELISA using either 3H5 or 4G2 mAbs. Glycosylation of recombinant DENV-2 E was assessed by ELISA and protein blot using Con A-HRPO conjugate. For both HRPO conjugates, TMB substrate was used for color development. To assess VLP formation, the purified protein (adjusted to a concentration of 5–10 µg/ml in 20 mM Tris-HCl, pH 8.5, containing 50 mM NaCl) was negatively stained with 1% uranyl acetate and examined under a Tecnai electron microscope as before [27].

### Mouse immunization

Immunizations were performed using groups of 6–8 weeks old Balb/C (n = 6), Swiss Albino (n = 6), and AG129 (n = 4–6) mice. The mice were immunized intra-peritoneally (i.p) with recombinant DENV-2 E VLPs. In all cases, immunization comprised three doses given on days 0, 30 and 90. Routine immunization dose was 20 µg antigen coated on 500 µg alum (in 100 µl 1× PBS). In some experiments, lower antigen doses were also tested. Sera were obtained ∼7–10 days after each boost for analysis of antibody titers. Balb/C and Swiss Albino immunizations were carried out at Abexome Biosciences (Bangalore, India). AG129 experiments were carried out at Ranbaxy Research Laboratories and ICGEB.

### Analysis of antibodies by ELISA and immunofluorescence assay

Antibody titers in murine sera were determined using indirect ELISAs with either recombinant protein antigens (DENV-2 E VLPs, EDIII-1, EDIII-2, EDIII-3 and EDIII-4) or with DENVs (DENV-1, DENV-2, DENV-3 and DENV-4) as coating antigens. The ability of antibodies induced by DENV-2 E VLPs to recognize DENV-2 was analyzed by immunofluorescence. Briefly, DENV-2-infected BHK-21 cells were fixed and probed with a pooled serum (1∶50 diluted) from DENV-2 E VLP-immunized Balb/C mice. Virus-bound antibodies in the DENV-2-infected cells were detected using secondary anti-mouse IgG-FITC conjugate and visualized by fluorescence microscopy.

### FACS based neutralization assay

A FACS-based virus neutralization assay that uses either Vero or DC-SIGN-expressing U937 cells [30] was used to determine DENV-neutralizing antibody titers (both homotypic as well as heterotypic) in the sera of immunized mice. The DENVs used in this assay were the WHO reference strains described earlier [30]. Virus-infected cells were identified using mAb 2H2-Alexa 488 conjugate. The serum dilution capable of causing a 50% reduction in the number of DENV-infected cells compared to that in the absence of immune serum is designated as FACS Neutralization Titer (FNT_50_).

### Assessment of protective efficacy of DENV-2 E VLP immunization

A challenge model based on AG129 mouse was developed using a previously published method [35]. To set up this model, an Indian DENV-2 isolate was passaged alternately between C6/36 cells and AG129 mice through multiple cycles. During the course of these cycles the virus was tested on AG129 mice for its potential to induce signs of sickness and cause death (Figure S1). Once an adequate stock of virulent virus was obtained, a dose-response experiment was set up to identify a dose that resulted in 100% lethality within a week post-challenge.

Groups of 6-8 weeks old AG129 mice were either mock-immunized (n = 4) or immunized with 20 µg purified DENV-2 E VLPs (n = 6) formulated in alum (i.p., days 0, 30 and 90). Ten days after the final immunization (day 100) each mouse was administered (i.p.) 1.4×10^8^ PFU of the challenge DENV-2 strain produced as described above. The mice were monitored twice daily for clinical symptoms and mortality for up to 18 days. At the end of the experiment the data were used to generate Kaplan Meier survival curves and analyzed by the log rank test (Mantel-Cox) for statistical significance using GraphPad Prism software.

Additional details relating to the methods above are available Protocol S1.

## Results

### Expression of *DENV-2 E* gene in *P. pastoris*

**Figure 1:**
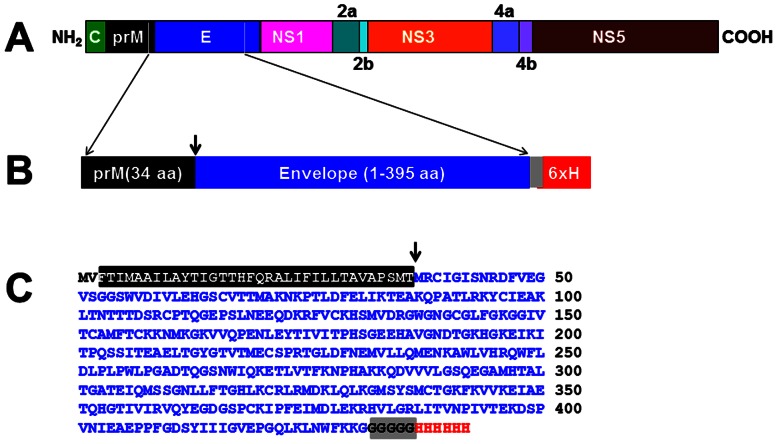
Design of the DENV-2 E antigen. (A) Schematic representation of the DENV-2 polyprotein, showing the parts of prM and E included in designing the E antigen for expression in *P. pastoris*. (B) Design of the DENV-2 E antigen consisting of the 395 aa residue E ectodomain, preceded by the C-terminal 34 aa residues of prM. The grey box denotes the pentaglycine linker peptide joining the C-terminus of E ectodomain to the polyhistidine tag (6×H). (C) The predicted aa sequence of the DENV-2 E antigen shown in ‘B’. The color scheme corresponds to that shown in ‘B’. The first two aa residues (MV) were introduced due to the insertion of the initiator codon in a Kozak consensus context. The downward arrows in ‘B’ and ‘C’ denote the signal cleavage site.

**Figure 2:**
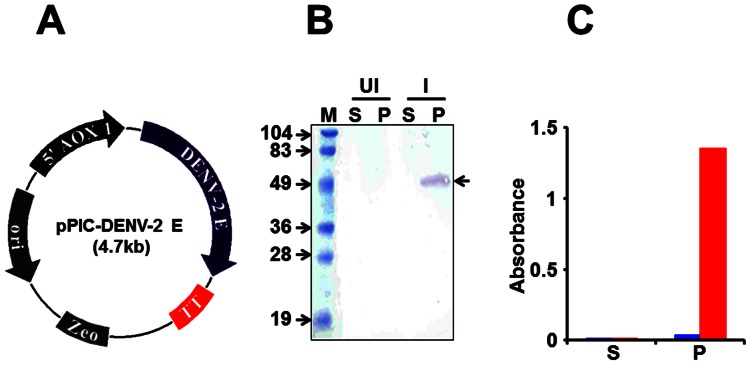
Expression of DENV-2 E in *P. pastoris*. (A) Map of the DENV-2 expression construct for integration into *P. pastoris* genome. The *DENV-2 E* gene is flanked by the *AOX1* promoter (5’ AOX1) and transcription terminator (TT) at its 5′ and 3′ ends, respectively. The construct contains an *E. coli* origin of replication (ori) and the selection marker zeocin (Zeo), which is functional in both *E. coli* as well as *P. pastoris*. (B) Localization of the recombinant DENV-2 antigen expression in induced *P. pastoris*. Aliquots of un-induced (UI) and induced (I) cultures were lysed and separated into soluble (S) and membrane-enriched pellet (P) fractions, run on SDS-polyacrylamide gel and subjected to Western blot analysis using mAb 24A12. Pre-stained protein markers were analyzed in lane ‘M’. Their sizes (in kDa) are indicated to the left. The arrow on the right indicates the position of the recombinant DENV-2 E antigen. (C) Ni-NTA His-Sorb ELISA analysis of S and P fractions obtained from UI (blue bars) and I (red bars) cell lysates described in ‘B’.

We designed a synthetic gene, *DENV-2 E*, codon-optimized for expression in *P. pastoris* (Figure 1). This gene was placed under the *AOX1* promoter of *P. pastoris* to create the expression vector, pPIC-DENV-2 E, shown in Figure 2A. This vector was integrated into the genome of the host *P. pastoris* strain KM71H. Results from a typical methanol-induction experiment performed using one of the resultant *DENV-2 E* gene-harboring *P. pastoris* clones is shown in Figure 2B. In this experiment, lysates were prepared from yeast cells, either before or after methanol induction, and separated into S and membrane-enriched P fractions. The presence of recombinant DENV-2 E protein in these fractions was detected by SDS-PAGE followed by immunoblotting with DENV-2 EDIII-specific mAb 24A12. This mAb identified a protein, with mobility consistent with that predicted for recombinant DENV-2 E, which appeared upon methanol-induction. Further, it was detectable only in the P fraction. This observation was also corroborated using a mAb specific to the 6×His affinity tag (data not shown). An ELISA wherein the recombinant protein was captured in Ni^2+^-NTA coated microtiter wells and revealed using mAb 24A12 (in conjunction with a secondary antibody enzyme conjugate), revealed the recombinant DENV-2 E antigen to be associated predominantly with the P fraction, as shown in Figure 2C. Next, we sought to optimize the induction conditions so that we may maximize recombinant DENV-2 E expression. Based on this (Figure S2), induction for the purpose of purification was carried out using 0.5% methanol, added every 12 hours, for a period of 3 days.

### Purification of *P. pastoris*-expressed recombinant DENV-2 E protein

**Figure 3:**
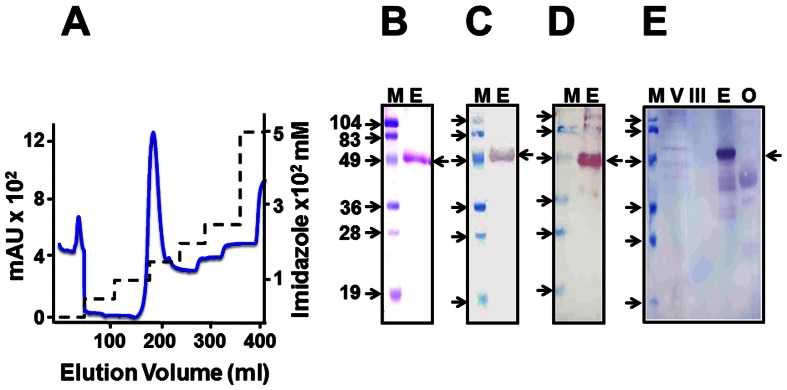
Purification and characterization of recombinant DENV-2 E antigen. (A) Ni^2+^ affinity chromatographic purification of DENV-2 E antigen from the P fraction of induced *P. pastoris* lysate. The continuous blue and the dashed black curves represent the profiles of UV absorbance (at 280 nm) and the imidazole step gradient, respectively, during chromatography. (B) Coomassie-stained SDS-polyacrylamide gel analysis of the purified protein. (C) Immunoblot analysis of the purified protein using mAb 24A12. (D) Immunoblot analysis of the purified protein using penta-His mAb. (E) Protein blot using Con A-HRPO conjugate. Controls analyzed in parallel include DENV-2 (lane ‘V’), purified EDIII-2 protein (lane ‘III’) and ovalbumin (lane ‘O’). In panels B-E: lanes ‘E’ denote the purified DENV-2 E protein (pooled peak material shown in panel ‘A’). Protein markers (whose sizes, in kDa, are shown to the left of each panel) were run in lanes ‘M’. The arrow to the right of each panel indicates the position of the recombinant DENV-2 E antigen.

Affinity purification using the engineered 6×His tag was performed under denaturing conditions as the *P. pastoris*-expressed recombinant antigen was predominantly associated with the insoluble P fraction. Expression of recombinant DENV-2 E protein was induced under conditions optimized above and the resultant biomass lysed under native conditions to obtain the membrane-enriched P fraction. This fraction, which served as the starting material for purification, was solubilized under denaturing conditions. Using His-Sorb ELISA, we found that the efficiency of extraction using 6 M GuHCl was approximately twice as much as that obtained with 8 M urea (data not shown). However, GuHCl is not compatible with subsequent SDS-PAGE analysis of the column fractions. Therefore, after binding to Ni^2+^-NTA affinity matrix, the chromatographic column was washed extensively with 8 M urea-containing buffer and eluted using a step gradient of imidazole. A single major peak eluted at 150 mM imidazole as shown in Figure 3A. An SDS-PAGE analysis of column fractions across this peak revealed a single major protein band of the predicted size. This protein band in all these peak fractions was also recognized by mAb 24A12 in a Western blot (Figure S3). The peak fractions were pooled, dialyzed, and an aliquot analyzed on SDS-PAGE, as shown in Figure 3B. This pooled material was also tested in Western blots and found to be recognized by mAb 24A12 (Figure 3C) and a mAb specific to the 6×His affinity tag (Figure 3D). The purified protein was also recognized by the conformation sensitive mAbs 3H5 and 4G2 in ELISA (data not shown). A protein blot using Con A-HRPO showed the purified protein to be glycosylated (Figure 3E). Binding of DENV-2 E to Con A was also corroborated in an ELISA format (data not shown). Based on densitometric analysis of the blot in Figure 3B, we estimate the purity of the pooled and dialyzed recombinant DENV-2 E to be ∼95%. We routinely obtained ∼15 mg purified recombinant DENV-2 E protein per liter of starting culture (data not shown).

As indicated earlier, we had included a 34 aa signal peptide sequence from the C-terminal end of prM to ensure proper processing of the E protein. As the difference between the processed and unprocessed forms is ∼4 kDa, it is not possible to determine unambiguously if processing had occurred based on SDS-PAGE mobility. Therefore, we analyzed the N-terminal aa sequence of two batches of the purified DENV-2 E antigen. This analysis revealed that processing had indeed occurred (data not shown) at the native signal peptidase cleavage site (indicated by the downward arrow in Figures 1B and 1C).

### *P. pastoris*-expressed DENV-2 E possesses inherent capacity to form VLPs

**Figure 4:**
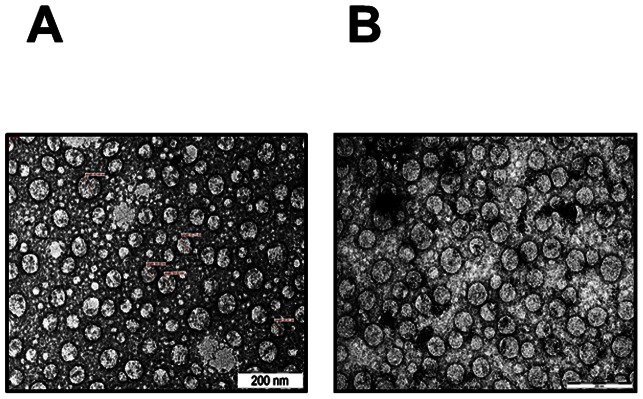
Electron microscopic analysis of purified DENV-2 E protein. (A) Freshly purified DENV-2 E antigen was negatively-stained with uranyl acetate and examined under EM. (B) EM analysis was carried out after incubating the purified antigen at 37°C for 2 weeks.

It has been reported that co-expression of prM and E proteins of flaviviruses including DENVs [14]-[17] in eukaryotic host systems leads to assembly of these two proteins into VLPs. We had observed in the past that *P. pastoris*-expressed DENV-2 E ectodomain formed VLPs when fused to the N-terminus of Hepatitis B virus surface antigen (HBsAg) [36]. Surprisingly, we found that deletion of the HBsAg carrier did not abrogate VLP-forming ability of DENV-2 E. EM analysis of the purified recombinant DENV-2 E protein preparation contained discrete VLPs, ranging in size from 20-40 nm, as shown in Figure 4A. As the recombinant antigen was purified under denaturing conditions, the presence of VLPs in the purified preparation indicates that assembly of monomeric DENV-2 antigen presumably occurred concomitant with gradual removal of urea during dialysis. The integrity of these VLPs remained essentially intact upon storage at 37°C for 2 weeks. In fact, the data reveal a greater proportion of VLP homogeneity (Figure 4B). This may be a reflection of a gradual VLP maturation event whereby greater homogeneity is achieved, akin to a similar phenomenon reported for hepatitis B VLPs [37].

### Recombinant DENV-2 E VLPs elicit virus-specific antibodies

**Figure 5:**
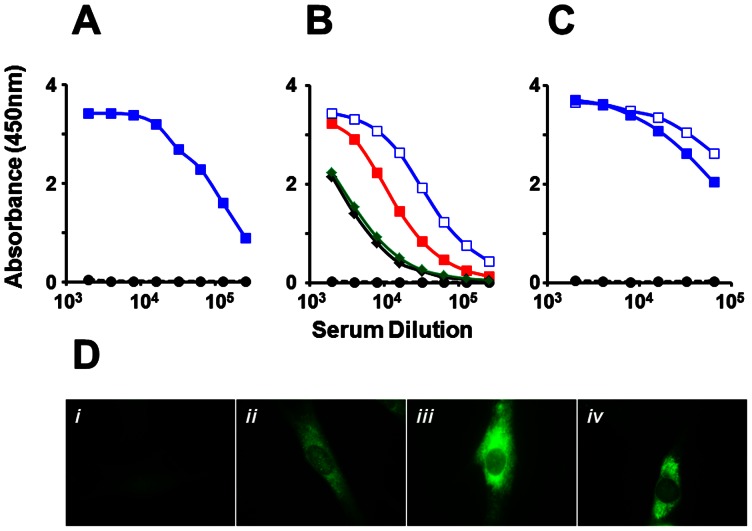
Evaluation of antibodies elicited by recombinant DENV-2 E antigen VLPs. (A) Pooled sera from DENV-2 E immunized (solid, blue curve) and mock-immunized (black, dashed curve) Balb/C mice were tested in an indirect ELISA using DENV-2 E protein as the coating antigen. (B) The Balb/C anti-DENV-2 E antiserum (panel A) was tested in ELISAs using recombinant monovalent EDIII-1 (black), EDIII-2 ( blue), EDIII-3 (red) or EDIII-4 (green) antigens. Mock-immunized Balb/C serum was tested against EDIII-1 as coating antigen (black, dashed). (C) Pooled serum from DENV-2 E-immunized Swiss albino mice was tested in ELISAs using either recombinant DENV-2 (solid blue squares) or EDIII-2 (empty blue squares) as the coating antigens. Mock-immunized Swiss albino serum was tested against EDIII-2 as coating antigen (black, dashed). (D) Indirect immunofluorescence analysis of DENV-2-infected BHK cells using mock-immunized serum (i), anti-EDIII-2 antiserum (ii), 4G2 mAb (iii), or anti-DENV-2 E antiserum (iv), as the source of primary antibodies. Bound antibodies were visualized using anti-mouse IgG-FITC conjugate. Antisera in panel (i), (ii) and (iv) were from Balb/C mice.

**Figure 6:**
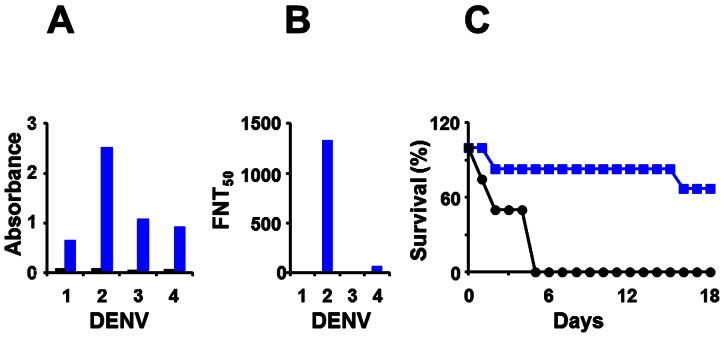
Characterization of DENV-2-specific antibodies elicited by DENV-2 E VLPs. (A) Analysis of virus-specific antibody titers in anti-DENV-2 E antisera (blue bars) and mock-immune sera (black bars) in indirect ELISAs using infectious DENVs as coating antigen. (B) Determination of virus-neutralizing antibody titers using FACS neutralization assay. Serial dilutions of anti-DENV-2 E antisera were tested for their capacity to neutralize infectivity of all four DENV serotypes [30]. The vertical axis denotes the serum dilution corresponding to 50% neutralization (FNT_50_ titre) of virus infectivity. Murine sera used in experiments shown in panels A and B were from Balb/C mice; the Arabic numerals along the x-axis, in both these panels, indicate DENV serotype. (C) Determination of protective efficacy of DENV-2 E VLP immunization. AG129 mice were either mock-immunized (black curve, n = 4) or immunized with DENV-2 E VLPs (blue curve, n = 6) and challenged with a virulent strain of DENV-2. The mice were monitored daily (up to 18 days post challenge) for mortality and the resultant data plotted as Kaplan-Meir survival curves.

Next, we investigated the immunogenicity of the DENV-2 E VLPs above. Balb/C mice were immunized i.p. with 20 µg dose of DENV-2 E VLPs formulated in alum using a three-dose regimen as described in ‘Methods’, based on preliminary experiments to assess the effect of antigen dose and boosting on antibody titers (Figure S4). Immune sera were analyzed in an indirect ELISA using purified DENV-2 E VLP as the coating antigen, as shown Figure 5A. This revealed the DENV-2 E VLPs purified from *P. pastoris* to be highly immunogenic. The high immunogenicity was also evident when we substituted recombinant EDIII-2, instead of DENV-2 E VLPs, as the coating antigen, suggesting that the EDIII of DENV-2 E VLPs is freely accessible to anti-DENV-2 antibodies. In addition, we also observed that anti-DENV-2 E antibodies also manifested cross-reactivity towards EDIII antigens corresponding to the remaining three DENV serotypes (Figure 5B). Cross reactive antibody titers were approximately 20% (EDIII-1 and EDIII-4) to 50% (EDIII-3) of the antibody titers specific to EDIII-2. This is consistent with the presence of cross-reactive epitopes in EDIII [38]. The high immunogenicity of recombinant DENV-2 E VLPs seen in Balb/C was also corroborated using Swiss albino mice, as shown in Figure 5C. Once again, this was evident using either DENV-2 E VLP or EDIII-2 as the coating antigen. We then tested if the antibodies elicited by DENV-2 E VLPs could recognize and bind to DENV-2. For this purpose, we carried out an indirect immunofluorescence assay, shown in Figure 5D. In this experiment, antibodies in DENV-2 E VLP-immunized Balb/C sera recognized DENV-2 infected BHK cells, far more efficiently than antisera raised against recombinant EDIII-2 (compare panels ii and iv, Figure 5D). The observed pattern of fluorescence is consistent with cytoplasmic replication of DENVs in distinct membrane associated zones. Having shown that recombinant DENV-2 E VLPs elicit virus-specific antibodies, we next assessed the relative homologous versus heterologous virus-specific antibody titers. Once again, we used the same indirect ELISA format as above, using DENV-1, DENV-2, DENV-3 or DENV-4 as the coating antigen instead of the recombinant protein antigens. These data, summarized in Figure 6A reveal that heterologous virus-specific antibody titers were ∼25–50% lower compared to homologous DENV-2-specific antibody titers, a trend mirroring the pattern observed using recombinant EDIIIs of the four DENV serotypes, as coating antigens (Figure 5B).

### Anti-DENV-2 E VLP antibodies are potent neutralizers of DENV-2 infectivity

As antibodies elicited by *P. pastoris*-expressed recombinant DENV-2 E VLPs manifested the ability to recognize and bind infectious DENV-2, we addressed if these antibodies may also have the potential to block the virus infectivity in a FACS-based virus-neutralization assay using Vero cells [30]. This assay, the results of which are depicted in Figure 6B, revealed that recombinant DENV-2 E VLP-induced antibodies neutralized DENV-2 with FNT_50_ titers >1200. Interestingly, the neutralizing activity appeared to be predominantly homotypic, with virtually no effect on DENVs-1 and -3 and minimal neutralizing effect on DENV-4. When we performed the FACS-based neutralization assay using U937 cells engineered to express DC SIGN, DENV-2-specific FNT_50_ titers were found to be ∼400. As these cells also carry Fc receptors which can mediate DENV entry, the observed titers presumably reflect the net outcome of the dynamic balance between neutralization and enhancement. Importantly, this DENV-2 neutralizing activity conferred significant protection upon AG129 mice challenged with a lethal dose of the virulent DENV-2 strain, as shown in Figure 6C (p<0.05). The challenge DENV-2 strain was developed in-house (Figure S1 and Protocol S1), by adapting a previously reported method of alternate passaging between C6/36 cells and AG129 mice [35].

## Discussion

A preventive vaccine for dengue continues to be an unmet need. Experience with LAVs which are the front runners in the dengue vaccine development pipeline has identified significant challenges. A major hurdle in dengue LAV development is the difficulty in obtaining a balanced immune response against all four DENVs due to interactions between the monovalent vaccine viruses when administered as a tetravalent formulation [8], [11], [12]. Recent data from the tetravalent CYD vaccine proof-of-concept trial in Thailand which revealed a near total lack of efficacy against DENV-2 [39], has served to underscore the importance of exploring and accelerating non-replicating dengue vaccine development efforts [8]. In this regard, genome-free dengue VLPs offer a potentially promising alternative.

DENV E-containing VLPs produced using *P. pastoris* have been documented in the literature (Table S1). However, the only instances are when it is co-expressed with prM [14], [15] or prM+C [40]. In the current work, we have addressed the question whether recombinant DENV-2 E by itself would possess VLP-forming potential. We expressed recombinant DENV-2 E (lacking the C-terminal 100 aa residues of full-length E) in *P. pastoris* and found it to be associated exclusively with the insoluble membrane-enriched P fraction, consistent with earlier studies on C-terminally deleted variants of DENV-2 E [41] and DENV-4 E [42], [43]. We purified the protein under denaturing conditions using a single affinity chromatographic step achieving ∼95% purity and ∼15 mg/L yield. Amino-terminal sequence analysis showed that the prM-derived signal peptide was cleaved off efficiently. Also, the protein was found to be glycosylated. Importantly, we observed that the recombinant DENV-2 E antigen in the purified preparation contained discrete VLPs. That this is an inherent attribute of the DENV-2 E antigen, that happened post-expression, is suggested by the observation that the protein was purified under strongly denaturing conditions. Assembly into VLPs presumably occurred upon gradual removal of the denaturant. This notion is consistent with recent ultrastructural and immunocytochemical analyses which strongly suggest that *P. pastoris*-expressed HBsAg antigen assembles into VLPs *in vitro* during downstream processing [44]. It is interesting to note that recombinant E ectodomain antigens expressed in baculovirus-infected *Sf*21 cells [45] or stably transfected *Drosophila* S2 cells [46] do not form VLPs. Clearly, there are differences between the insect and yeast expression systems, such as difference in glycosylation, which we do not understand completely.

These VLPs were immunogenic when tested in both inbred and outbred mice eliciting DENV-2 virus-specific antibodies. Using a FACS-based virus neutralization assay, we observed that the DENV-2 E VLP-induced antibodies neutralized virus infectivity very efficiently (FNT_50_ titers >1∶1200). Importantly, this response was predominantly homotypic with no cross-reactivity towards DENV-1 and DENV-3 and minimal cross-neutralizing titers to DENV-4. The basis for this observation needs to be explored. Recent data from Sanofi’s CYD vaccine trial have raised questions about the validity of using Vero cells for virus-neutralization assays, suggesting that the use of Fc receptor-bearing cells to measure neutralizing antibody titers may be more relevant in assessing the vaccine’s immunogenicity [39]. Repeating the neutralization assay using U937 cells engineered to express DC-SIGN resulted in DENV-2-specific FNT_50_ titers of ∼400. The lower neutralization titers observed with U937-DC-SIGN cells suggests DENV-2 entry into cells through Fc receptors as well. Importantly, we found in a preliminary experiment that immunization with DENV-2 E VLP partially protected AG129 mice against lethal challenge with a virulent DENV-2 strain. The level of protection, however, was significant (p<0.05). Further experiments are on to optimize antigen dose, immunization schedule, and challenge virus dose to obtain additional data to assess the protective efficacy of DENV-2 E VLPs.

In conclusion, we have shown that recombinant DENV-2 E ectodomain expressed in *P. pastoris* undergoes post-translational processing, enabling it to fold in a way that permits its self-assembly into VLPs, in the absence of prM co-expression. Further, these DENV-2 E VLPs can be produced at high levels and possess excellent immunogenicity in different strains of mice, capable of eliciting high titer neutralizing antibodies, using both epithelial and Fc receptor-bearing cell substrates and capable of conferring partial protection against lethal DENV-2 challenge in a mouse model. The role, if any, of the DENV-2 E VLP-induced antibodies in ADE needs to be evaluated. The data presented in this work strongly warrant further exploration of this approach towards developing a tetravalent dengue VLP vaccine using the *P. pastoris* expression system. Work is underway to delete the 6× His tag and devise a purification strategy based on conventional chromatographic methods, so that we may extend this approach to the remaining three DENV serotypes to make a tetravalent formulation. This will have the advantage of fine tuning the immune response to each serotype, if necessary, through modification of the relative proportions of the four monovalent VLPs.

## Supporting Information

Figure S1 **Evaluation of the challenge virus.** Panel ‘*a*’ shows healthy AG129 mice. Panels ‘*b*’ and ‘*c*’ show infected mice manifesting ruffled fur, hunched back and hind limb paralysis (day 3 post-challenge). These were administered (i.p.) 1.4×10^8^ PFU each of the in-house developed DENV-2 challenge virus (described in Protocol S1). Panel ‘*d*’ shows mice that succumbed to virus challenge (day 5 post-challenge).
(TIF)

Figure S2 **Optimization of induction of DENV-2 E expression.** (A) *P. pastoris* clone harboring the DENV-2 E gene expression construct was induced at logarithmic phase of growth with 1% methanol, followed by withdrawal of aliquots at 6 (lane 1), 12 (lane 2), 24 (lane 3), 48 (lane 4), 72 (lane 5), 96 (lane 6) and 120 (lane 7) hours post-induction. Analysis of these samples by Western blot is shown on the top and His-Sorb ELISA on the bottom. (B) Multiple parallel small-scale cultures of the *P. pastoris* clone (described in ‘A’) were set up and each one was induced separately with 0.5, 1 or 2% methanol for 72 hours. As in panel A, the top and bottom parts show the Western blot and His-Sorb ELISA results, respectively. All inductions beyond 12 hour duration were maintained by the addition of methanol, at the appropriate concentration, at 12 hour intervals. In the Western blots, ‘M’ and ‘U’ correspond to lanes in which protein size markers and un-induced lysates were analyzed. The sizes of the markers (in kDa) are shown to the left of the blots; the arrow on the right indicated the position of the recombinant protein.
(TIF)

Figure S3 **Analysis of Ni^2+^-NTA peak elution fractions.** (A) SDS-PAGE analysis of fractions (lanes 1–6) across the major peak (eluting at 150 mM imidazole) shown in Figure 3A. Separated protein bands were visualized by Coomassie staining. (B) Western bot analysis of the same peak fractions, analyzed in pane ‘A’. After electrophoresis, separated proteins were transferred to a nitrocellulose membrane and probed using mAb 24A12 in conjunction with anti-mouse IgG-HRPO plus TMB substrate. In both panels, protein size markers were analyzed in lanes marked ‘M’. Their sizes (in kDa) are shown to the left of the panels; the arrow to the right of each panel indicates the position of the purified recombinant DENV-2 E protein.
(TIF)

Figure S4 **Preliminary investigation of the immunogenicity of recombinant DENV-2 E VLPs.** (A) Analysis of boosting effect. Balb/C mice were immunized with DENV-2 E VLPs (20 µg formulated in alum) on days 0, 30 and 90. Sera were collected after the first (empty blue squares) and the second (filled blue squares) boosts, on days 37 and 100, respectively and tested for antibody titers in indirect ELISA. (B) Determination of antigen dose. Balb/C mice were immunized with 2 µg (green), 6 µg (red) or 20 µg (blue) of DENV-2 E VLPs, formulated in alum, following the same immunization schedule as in ‘A’. Sera collected after the 2^nd^ boost (day 100) were analyzed in ELISA as before. In both panels A and B, sera from mock-immunized mice were analyzed in parallel (dashed black curves); in both experiments, the coating antigen was purified DENV-2 E VLPs.
(TIF)

Table S1 **DENV antigens expressed using *P. pastoris*.**
(DOCX)

Protocol S1 **Supplementary protocol details.**
(DOCX)
