# Supplementary figures and images for: Pichia pastoris-Expressed Dengue 2 Envelope Forms Virus-Like Particles without Pre-Membrane Protein and Induces High Titer Neutralizing Antibodies
Source: PLoS One. 2013 May 23;8(5):e64595. doi: 10.1371/journal.pone.0064595 (PMC3662778; doi:10.1371/journal.pone.0064595)

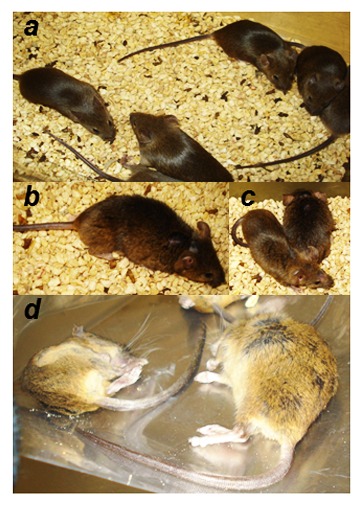

Supplement: Figure S1 — Evaluation of the challenge virus. Panel ‘a’ shows healthy AG129 mice. Panels ‘b’ and ‘c’ show infected mice manifesting ruffled fur, hunched back and hind limb paralysis (day 3 post-challenge). These were administered (i.p.) 1.4×10^8^ PFU each of the in-house developed DENV-2 challenge virus (described in Protocol S1). Panel ‘d’ shows mice that succumbed to virus challenge (day 5 post-challenge). (TIF) [file pone.0064595.s001.tif]

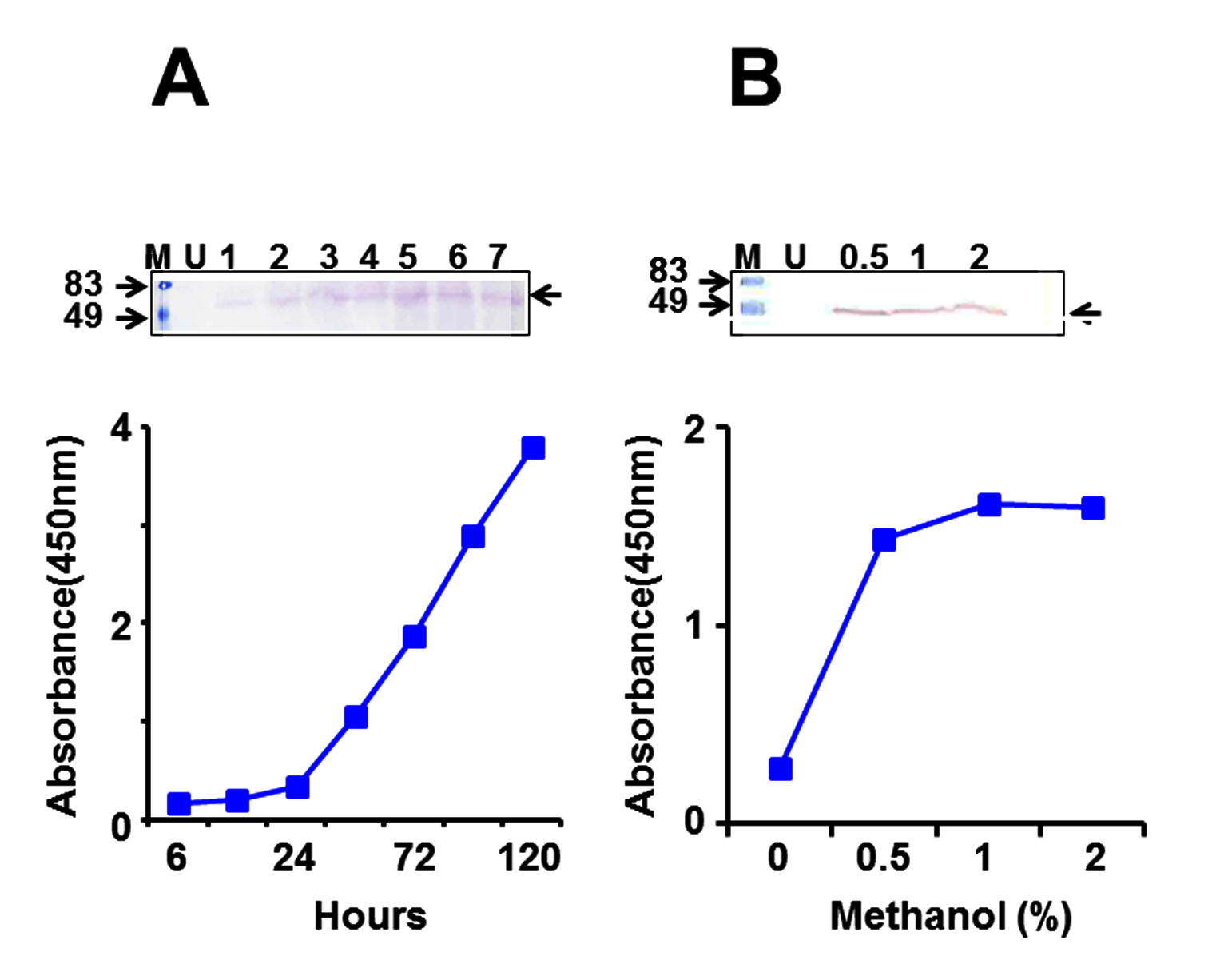

Supplement: Figure S2 — Optimization of induction of DENV-2 E expression. (A) P. pastoris clone harboring the DENV-2 E gene expression construct was induced at logarithmic phase of growth with 1% methanol, followed by withdrawal of aliquots at 6 (lane 1), 12 (lane 2), 24 (lane 3), 48 (lane 4), 72 (lane 5), 96 (lane 6) and 120 (lane 7) hours post-induction. Analysis of these samples by Western blot is shown on the top and His-Sorb ELISA on the bottom. (B) Multiple parallel small-scale cultures of the P. pastoris clone (described in ‘A’) were set up and each one was induced separately with 0.5, 1 or 2% methanol for 72 hours. As in panel A, the top and bottom parts show the Western blot and His-Sorb ELISA results, respectively. All inductions beyond 12 hour duration were maintained by the addition of methanol, at the appropriate concentration, at 12 hour intervals. In the Western blots, ‘M’ and ‘U’ correspond to lanes in which protein size markers and un-induced lysates were analyzed. The sizes of the markers (in kDa) are shown to the left of the blots; the arrow on the right indicated the position of the recombinant protein. (TIF) [file pone.0064595.s002.tif]

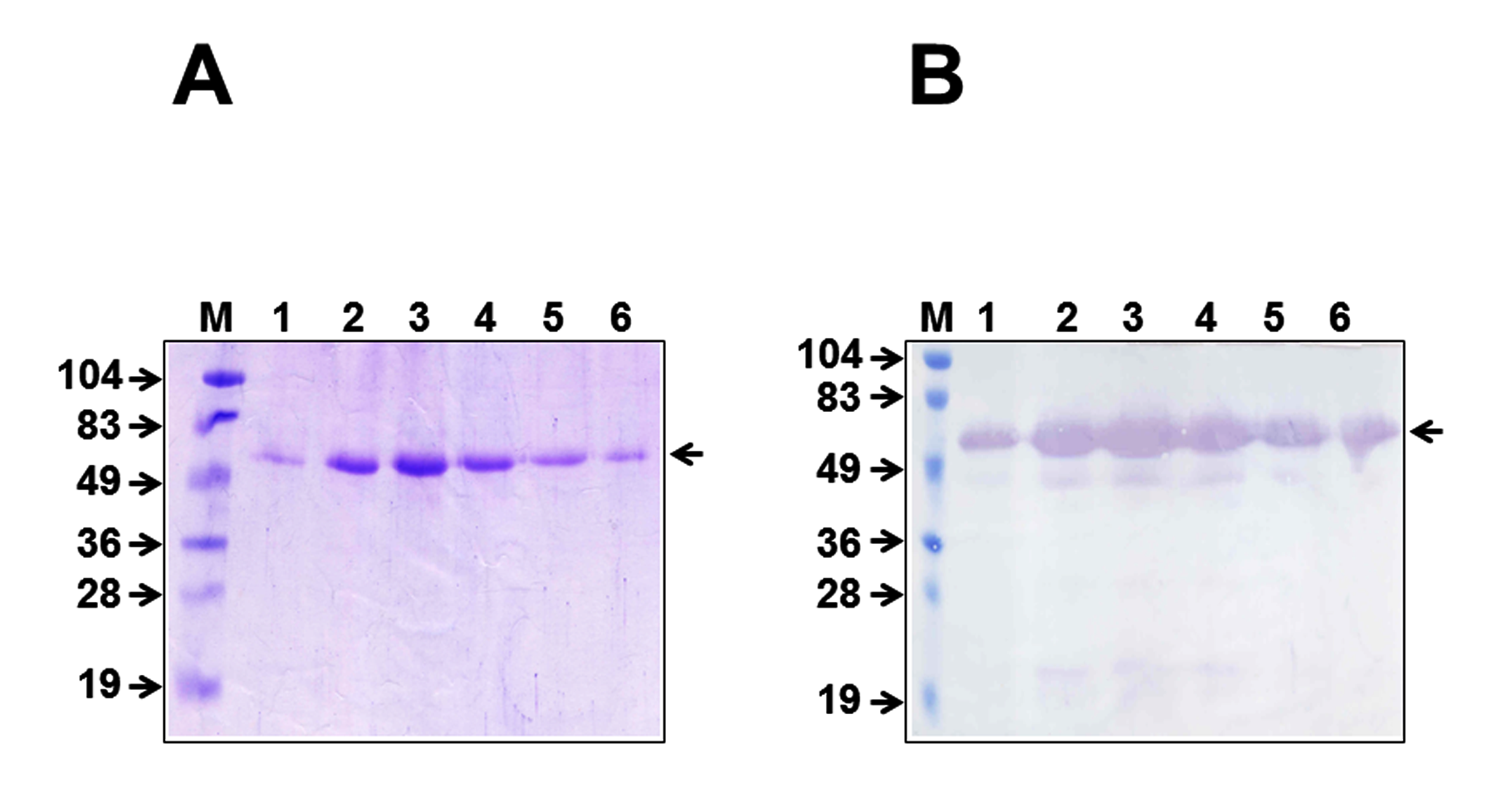

Supplement: Figure S3 — Analysis of Ni2+-NTA peak elution fractions. (A) SDS-PAGE analysis of fractions (lanes 1–6) across the major peak (eluting at 150 mM imidazole) shown in Figure 3A. Separated protein bands were visualized by Coomassie staining. (B) Western bot analysis of the same peak fractions, analyzed in pane ‘A’. After electrophoresis, separated proteins were transferred to a nitrocellulose membrane and probed using mAb 24A12 in conjunction with anti-mouse IgG-HRPO plus TMB substrate. In both panels, protein size markers were analyzed in lanes marked ‘M’. Their sizes (in kDa) are shown to the left of the panels; the arrow to the right of each panel indicates the position of the purified recombinant DENV-2 E protein. (TIF) [file pone.0064595.s003.tif]

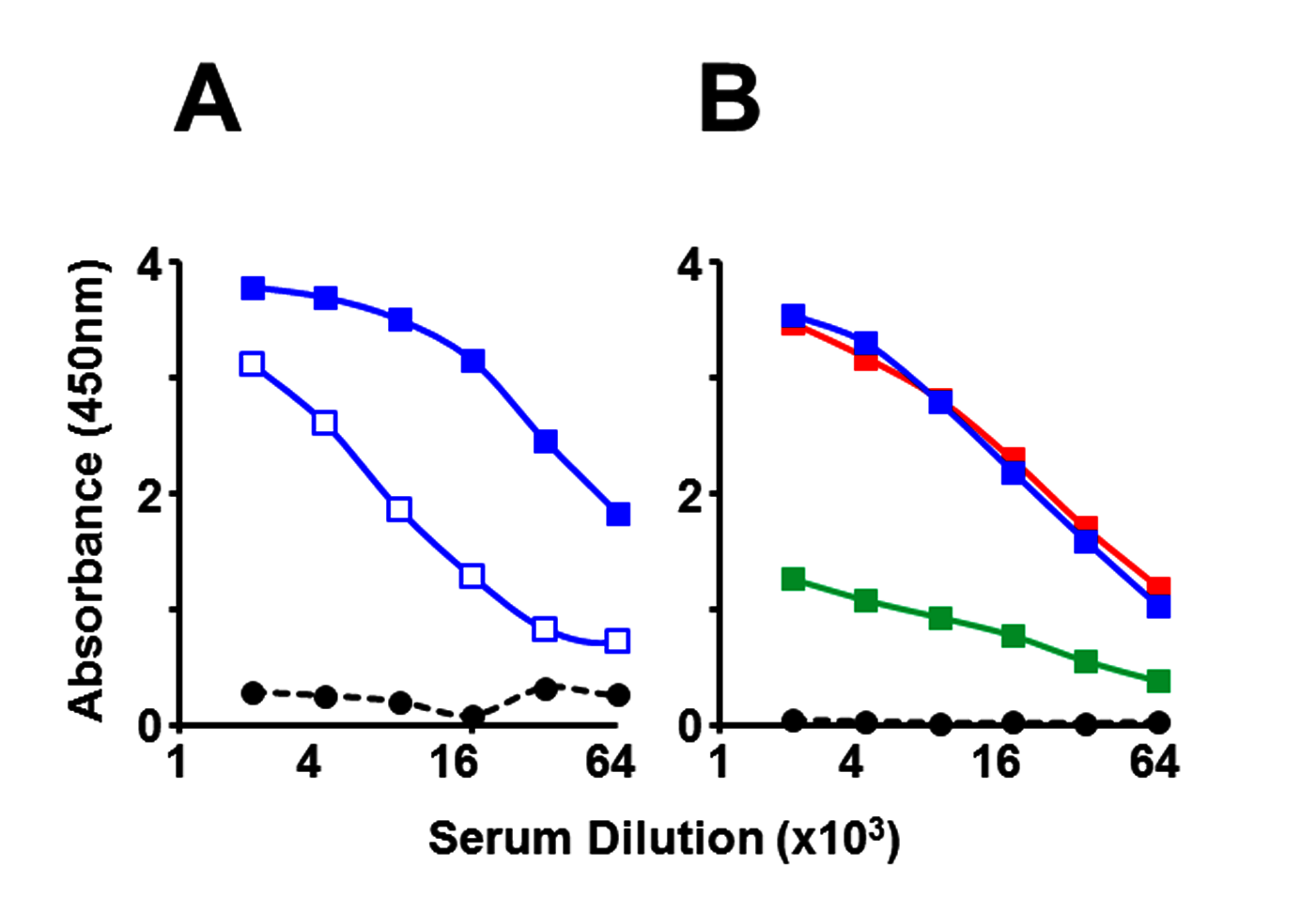

Supplement: Figure S4 — Preliminary investigation of the immunogenicity of recombinant DENV-2 E VLPs. (A) Analysis of boosting effect. Balb/C mice were immunized with DENV-2 E VLPs (20 µg formulated in alum) on days 0, 30 and 90. Sera were collected after the first (empty blue squares) and the second (filled blue squares) boosts, on days 37 and 100, respectively and tested for antibody titers in indirect ELISA. (B) Determination of antigen dose. Balb/C mice were immunized with 2 µg (green), 6 µg (red) or 20 µg (blue) of DENV-2 E VLPs, formulated in alum, following the same immunization schedule as in ‘A’. Sera collected after the 2nd boost (day 100) were analyzed in ELISA as before. In both panels A and B, sera from mock-immunized mice were analyzed in parallel (dashed black curves); in both experiments, the coating antigen was purified DENV-2 E VLPs. (TIF) [file pone.0064595.s004.tif]
