# Supplementary material for: Pichia pastoris-Expressed Dengue 2 Envelope Forms Virus-Like Particles without Pre-Membrane Protein and Induces High Titer Neutralizing Antibodies
Source: PLoS One. 2013 May 23;8(5):e64595. doi: 10.1371/journal.pone.0064595 (PMC3662778; doi:10.1371/journal.pone.0064595)
Supplement: Table S1 — DENV antigens expressed using P. pastoris. (DOCX) [file pone.0064595.s005.docx]

**Table S1: DENV antigens expressed using *P. pastoris***

| **Serotype** | **Antigen**^a^ | **Signal^b^** | **Signal**  **cleavage** | ***P. pastoris* Host**^c^ | **VLP** | **Neut titer**^d^ | **Efficacy**^e^ | **Reference**^f^ |
| --- | --- | --- | --- | --- | --- | --- | --- | --- |
| DENV-1 | GST-E (401) | None | - | GS115 (*his4*) | - | nd | nd | Sugrue *et al*, 1997 (JVM) |
| DENV-1 | C+prM+E | None | No | GS115 (*his4*) | Yes | 1:10 | nd | Sugrue *et al*, 1997 (JGV) |
| DENV-2 | E (395) HBsAg* | None | - | GS115 (*his4*) | Yes | 0 | nd | Bisht *et al*, 2001; 2002 |
| DENV-4 | E (442) | MFα | No | MP36(*his3*) | - | 1:5 | nd | Hermida *et al*, 2002 |
|  |  |  |  |  |  | >1:10 | + (Balb/C) | Mune *et al*, 2003 |
|  |  |  |  |  |  | 1:80-1:640** | + (Monkey) | Guzman *et al*, 2003 |
| DENV-2 | E (395) | *SUC2* | No | MP36(*his3*) | - | nd | nd | Valdes *et al*, 2007 |
| DENV-2 | E (495) | MFα | Yes | X33 | - | nd | nd | Wei *et al*, 2003 |
| DENV-2 | prM+E | C-sig | Partial | X33^#^ | Yes | 1:45 | nd | Liu *et al*, 2010 |
| DENV-1 | prM+E | None | - | X33^#^ | Yes | 1:40 | nd | Tang *et al*, 2012 |
| DENV-2 | E (395)* | prM-sig | Yes | KM71H | Yes | 1:1200** | + (AG129) | Present work |

^a^C, prM and E represent the DENV capsid, pre-membrane and envelope protein, respectively; the numbers in parenthesis indicate the number of amino acid residues in the C-terminally truncated E protein variants; fusion partners were used in some cases; GST: gluthathione S transferase; HBsAg: hepatitis B surface antigen; all antigens except the two marked by asterisk (~95% purified) in this column were partially purified (30-70%); in addition, the genes for these two were codon-optimized for *P. pastoris* expression; the rest were RT-PCR amplified genes.

^b^MFα*: S. cerevisiae* mating factor α signal peptide; *SUC2*: *S. cerevisiae* invertase signal peptide; C-sig: signal peptide derived from C-terminus of C protein; prM-sig: signal peptide derived from C-terminus of prM protein.

^c^ All hosts utilized the methanol-inducible *AOX1* promoter for expression, except the two marked with the ‘#’, which used the constitutive glyceraldehyde phosphate dehydrogenase promoter.

^d^The titers represent the serum dilution at which virus infectivity is neutralized by 50%; in only two instances, marked by the double asterisks in this column, alum was used as the adjuvant; all others used Freund’s adjuvant.

^e^Efficacy was shown (indicated by the ‘+’ sign) based on protection against encephalitis after intracranial challenge (Balb/C), reduction in viremia after intravenous challenge (Monkey) or survival after intraperitoneal challenge (AG129); nd: not determined.

Sugrue *et al*, *J. Virol. Meth*. **69:** 159-169, 1997; Sugrue *et al*, *J. Gen. Virol.***78:** 1861-1866, 1997; Bisht *et al, Prot. Exp. Purif*. **23:** 84-96, 2001; Bisht *et al, J. Biotechnol*. **99:** 97-110, 2002; Hermida *et al, J. Biotechnol*. **94:** 213-216, 2002; Mune *et al, Arch. Virol.* **148:** 2267-2273, 2003; Guzman *et al, Am. J. Trop. Med. Hyg*. **69:** 129-134, 2003; Valdes *et al, Mol. Biotechnol*. **35:** 23-30, 2007; Wei *et al, J. Virol. Meth.* **109:** 17-23, 2003; *Liu et al, Virus Genes* **40:** 53-59, 2010; Tang *et al, Chin. Med. J.* **125:** 1986-1992, 2012.
