# Supplementary material for: Pichia pastoris-Expressed Dengue 2 Envelope Forms Virus-Like Particles without Pre-Membrane Protein and Induces High Titer Neutralizing Antibodies
Source: PLoS One. 2013 May 23;8(5):e64595. doi: 10.1371/journal.pone.0064595 (PMC3662778; doi:10.1371/journal.pone.0064595)
Supplement: Protocol S1 — Supplementary protocol details. (DOCX) [file pone.0064595.s006.docx]

**Protocol S1: Additional experimental details**

**Tissue culture**

Mammalian cells were maintained in Dulbecco’s Modified Eagle medium (DMEM), supplemented with 10% (v/v) heat-inactivated fetal calf serum (ΔFCS), in a humidified 10% CO_2_ incubator, at 37^o^C. The C6/36 mosquito cell line was maintained in Leibovitz L15 medium supplemented with 10% ΔFCS and 0.03% tryptose phosphate broth, at 28^o^C in a CO_2_-free incubator.

**Creation and identification of DENV-2 E-expressing *P. pastoris* clone**

The *DENV-2 E* gene encodes the ectodomain of DENV-2 E protein comprising the first 395 aa residues, preceded by a signal peptide (corresponding to the C-terminal 34 aa residues of the prM protein) to ensure proper post-translational processing. It also contains a C-terminal 6×His tag to facilitate its purification. This gene was cloned into *Eco* RI and *Not* I sites of pPICZ-A to create the expression vector pPIC-DENV-2 E. This was linearized with *Sac* I and integrated into the host genome of *P. pastoris* strain KM71H by electroporation, followed by selection on zeocin (500µg/ml)-containing plates, as recommended in the vendor’s Easyselect^TM^ *Pichia* expression manual. Resultant transformants were screened for integration of the DENV-2 E expression cassette by polymerase chain reaction (PCR)-screening of genomic DNA, using primers designed to anneal to the insert-flanking vector sequences, as previously described [Arora *et al*, 1998]. Zeocin-resistant, PCR-positive clones were expression-screened using DENV-2 E-specific mAb 24A12 in His-Sorb ELISA as described before [Batra *et al*, 2010], to choose the best-expressing clone for further work.

**Induction and extract preparation**

Yeast cultures were grown in buffered glycerol-containing medium (BMGY) at 30^o^C to logarithmic phase. For induction, cells were spun down and re-suspended in methanol-containing medium (BMMY). To establish optimal induction conditions, logarithmically growing cultures were induced (i) at a fixed methanol concentration (1%) for up to 5 days, or (ii) for a fixed time (3 days) using methanol concentrations up to 2%. In either case, methanol was added at 12 hourly intervals. Recombinant DENV-2 E was detected by Western blot analysis and relative quantitation performed using His-Sorb ELISA, both with mAb 24A12. Routine inductions were performed with 0.5% methanol (added every 12 hours) for a period of 3 days.

Induced cells (~100 OD_600_) were suspended in 500µl cell suspension buffer, CSB (50mM Tris-HCl (pH 8.5)/500mM NaCl) and extracted using glass beads by shaking in a thermomixer overnight (1400 rpm, 4^o^C). The extract was spun down and separated into supernatant (S) fraction and pellet (P). The latter was extracted in (500µl) 6M guanidine-HCl (GuHCl), at room temperature (RT) for 4 hours, and spun down to obtain GuHCl-solubilized P fraction. The S and P fractions were analyzed for the presence of the recombinant DENV-2 E antigen by Western blot assay. GuHCl-solubilized P fractions were subjected to tri-chloro acetic acid precipitation prior to Western blotting. Relative levels of recombinant DENV-2 E protein were assessed by His-Sorb ELISA using mAb 24A12 [Batra *et al*, 2010].

**Ni2+-affinity purification**

A 100ml starter culture in YPD (1% yeast extract/2% peptone/2% dextrose) medium was set up and allowed to grow for 16-18 hours. This was used to inoculate 3L BMGY medium and let grow to log phase (~20 OD_600_). At this point cells were spun down (5,000 rpm, 5 min, 4^o^C), rinsed with sterile water to remove glycerol and re-suspended in 600ml (~100 OD_600_) BMMY containing 0.5% methanol. Induction was carried out for 3 days (with 0.5% methanol replenishment at 12 hour intervals). At all stages, cultures were grown at 30^o^C in baffled shake flasks (250 rpm), maintaining a culture volume to total flask volume ratio of 1:4 to 1:5, to ensure adequate aeration.

Induced cell pellet (~100 g wet weight) was washed in sterile 1× phosphate buffered saline (PBS), re-suspended in 300ml CSB and lysed with glass beads in a Dynomill in 5-7 cycles (10 minutes/cycle, followed by 5 minutes off time). The resultant lysate (pooled with ~300 ml Dynomill wash) was spun down in a SLA1500 rotor (10,000 rpm, 2 hours, 4^o^C). The membrane-enriched P fraction obtained was dispersed into 500ml membrane extraction buffer MEB (CSB supplemented with 6M GuHCl and 30mM imidazole) and stirred 3-4 hours at RT. This extract was clarified by centrifugation (13,000 rpm, 1 hour, 4^o^C) and filtration (0.45µ) and bound to 25ml Ni^2+^-NTA resin (50% slurry, pre-equilibrated in MEB) in batch mode, overnight at RT. This was packed into a chromatographic column, washed with 10 bed volumes of MEB, followed by 10 bed volumes of modified MEB in which 6M GuHCl was replaced with 8M urea. Bound protein was eluted using a step imidazole gradient in 8M urea-MEB. Chromatography was carried out using an AKTA purifier system. Column fractions were analyzed by SDS-PAGE and purified peak fractions pooled. This material was dialyzed against 20mM Tris-HCl (pH 8.5) buffer containing 50mM NaCl. During this dialysis urea concentration was reduced to zero in a step-wise manner.

**Western blot analysis**

Protein-containing samples (crude extracts, S and P fractions, column fractions and purified protein preps) were subjected to SDS-PAGE. Separated proteins were electro-transferred (in presence of 25mM Tris, 192mM Glycine, 20% methanol) to a nitrocellulose membrane using a semi-dry blotting apparatus (14V, 40 min, RT). After transfer, the membranes were blocked (by incubating 2 hours at RT in 5% skim milk in 1× PBS+0.1% Tween-20), rinsed (once in 1× PBS+1% Tween-20), and probed with primary antibody (mAb 24A12 at 1µg/ml or penta-His mAb at 0.1µg/ml, prepared in 1× PBS+1% Tween-20 containing 2.5% skim milk). After 1 hour incubation, blots were washed (5 times in 1× PBS+0.1% Tween-20) and incubated for a further hour in secondary antibody (anti-mouse IgG-HRPO, 0.1µg/ml prepared in 1× PBS+1% Tween-20 containing 1.25% skim milk). Blots were washed as before and developed using TMB substrate.

**Glycosylation assays**

Glycosylation of *P. pastoris*-expressed DENV-2 E was assessed using Con A-HRPO in ELISA and protein blot formats. For ELISA, microtiter wells were coated with purified recombinant antigen (250ng/100µl/well) or DENV-2 [2 x 10^6^ plaque forming units (pfu) polyethylene glycol precipitated virus/100µl/well] overnight at 4^o^C. Wells were washed three times (1× PBS+0.05% Tween-20) and blocked (300µl of 1× PBS+ 3% BSA/well) for 2 hours at 37^o^C.This was followed by the addition of Con A-HRPO (final concentration of 2µg/ml in 1× PBS containing 1.5% BSA and 0.05% Tween 20,100µl/well) and incubating for 1 hour at 37^o^C. Wells were washed once again followed by addition of TMB substrate (100µl/well) and incubation for 30 minutes at 37^o^C. The reaction was stopped using 1N H_2_SO_4_ and absorbance read at 450nm. The protein blot was performed essentially as described above for the Western blots. Briefly, the nitrocellulose membrane after blocking and washing was probed directly with Con A-HRPO (2µg/ml of 1× PBS+1.5% BSA+ 0.05% Tween 20), followed by washing and color development with TMB substrate as before.

**Indirect ELISA to determine antibody titers**

Antibody titers in murine sera were determined using indirect ELISAs with either recombinant protein antigens (DENV-2 E VLPs, EDIII-1, EDIII-2, EDIII-3 and EDIII-4) or with DENVs (DENV-1, DENV-2, DENV-3 and DENV-4). In the case of recombinant proteins, coating was performed overnight using 250ng/well in 100µl 0.1M sodium bicarbonate buffer, pH 9.6. In the case of DENVs, coating was done using virus-infected culture supernatant (100µl/well).Coated wells were washed (in 1× PBS+0.1% Tween-20, three times), blocked (in 5% skim milk in 1× PBS+0.1% Tween-20, for 2 hours at 37^o^C) and used for ELISA. Wells were incubated with serial dilutions of the murine antisera (100µl/well) for 1 hour at 37^o^C, washed 4-5 times and incubated for an additional hour with anti-mouse IgG-HRPO conjugate (0.1µg/ml; 100µl/well). This was followed by washing as before and color development using TMB substrate (100µl per well), which was terminated after 30 minutes by addition of 1N H_2_SO_4_ (100µl per well). Absorbance was read at 450nm.

**Immunofluorescence assay**

BHK-21 cells on coverslips (placed in 6 well plates seeded with 64,000 cells/well), grown for 24 hours (in DMEM+5% ΔFCS) at 37^o^C in a 10% CO_2_ incubator were infected with DENV-2 (m.o.i. =0.2, 750μl/well in DMEM+2% Δ FCS). After 2 hours exposure to virus inoculum, wells were supplemented with 1.25 ml growth medium and returned to the incubator. At 36 hours post-infection, the infected monolayers were rinsed three times in 1× PBS, incubated in 4% formaldehyde (2ml/well) for 15 minutes, rinsed again and incubated for a further 15 minutes in ice-cold methanol (2ml/well). Formaldehyde/methanol-treated monolayers were rinsed three times in TNT buffer (50mM Tris, 500mM NaCl, 0.1% Tween 20) and blocked using TNT buffer containing 5% PVP+25% goat serum for 2 hours at 37^o^C. Monolayers were washed with TNT buffer. Coverslips were retrieved from the wells and incubated with 50μl murine antisera (diluted 1:50 in TNT buffer containing 1% PVP+1% goat serum) at 37^o^C for 1 hour. Coverslips were rinsed 6 times in TNT buffer and incubated with 50μl anti-mouse IgG-fluorescein conjugate (25 μg/ml prepared in antibody dilution buffer above) for 1 hour at RT in the dark. Once again coverslips were rinsed in TNT buffer, inverted on a drop of antifade reagent, and visualized at 100x magnification in a fluorescence microscope.

**FACS neutralization assay**

Vero cells seeded in 96-well plates (25,000 cells/200μl/well) were grown in DMEM+5% ΔFCS for 24 hours in the incubator (37^o^C, 10% CO_2_). Aliquots of DENV-1 (43,000pfu/40µl), DENV-2 (7000pfu/40µl), DENV-3 (12,000pfu/40µl) and DENV-4 (57,000pfu/40µl), pre-determined to infect 10-15% of cells (within the linear range of the dose response curve) were pre-incubated with serial two-fold dilutions (in DMEM+2% ΔFCS) of heat-inactivated (56^o^C/30 minutes) murine immune serum for 1 hour in the incubator. This DENV-antiserum mix (80μl/well) was added to Vero cells in the 96-well plate (after aspirating off culture medium). After 2 hours of exposure, the inoculum was removed, the monolayer rinsed once, and replaced with DMEM+2% ΔFCS (200μl/well) and returned to the incubator. In parallel, control infections using sera from mock (PBS)-immunized mice in the pre-incubation step, as well as infections without any pre-incubation step, were set up in parallel. All infections were in duplicates. At 24 hours post-infection, cell monolayers in the 96-well plates were rinsed with 1× PBS, trypsinized (25µl/well of a 0.05% stock enzyme solution) and transferred to corresponding wells in a 96-well U-bottom plate (to facilitate the subsequent centrifugation steps). Cells were centrifuged at RT (1,500 rpm, 5 minutes), washed twice with 1× PBS (150μl/well/wash), re-centrifuged as before and fixed with 4% formaldehyde (50μl/well) at RT for 10 minutes. Once again, cells were centrifuged (2,500 rpm, 5 minutes, RT), washed twice with permeabilization buffer (1×PBS containing 2.5% BSA, 0.02% NaN_3_ and 0.1% saponin, 150μl/well/wash), re-centrifuged and blocked (using permeabilization buffer containing 1% normal mouse serum) for 30 minutes at RT. The blocking solution was removed by centrifugation and the cells incubated with mAb 2H2-Alexa 488 conjugate (diluted 1:400 in blocking buffer; 60μl/well) for 1 hour at 37^o^C with gentle shaking. Cells were pelleted down (2,500rpm, 5minutes, RT), washed twice (with permeabilization buffer) and re-suspended in FACS buffer (1× PBS containing 0.5% BSA and 0.02% NaN_3_) and analyzed using BD FACS-Verse flow cytometer (BD Biosciences).

**Development of DENV-2 challenge virus**

The challenge virus was generated from an Indian clinical DENV-2 isolate which was passaged alternately between C6/36 cells and AG129 mice following a modification of a previously published method [Shresta *et al*, 2006]. The initial isolate was used to infect C6/36 cells in a T-25 flask for about 2 weeks, concentrated by ultracentrifugation (SW28 rotor, 20,000 rpm, 2hours, 4^o^C), re-suspended in minimal volume of 1× PBS, filter-sterilized and injected intra-cranially into an AG129 mouse. At 3 days post-infection, the mouse was euthanized, the brain removed and homogenized. This homogenate was clarified, sterile-filtered and used to inoculate C6/36 cells. One week later, the culture supernatant was concentrated and injected i.c. into a second AG129 mouse. The virus was carried through multiple cycles of C6/36 cells and AG129 mouse brain passaging. After 8^th^ cycle, we began to observe signs of virulence when injected i.p into AG129. Clinical symptoms were usually lethargy, ruffled fur, hunched back and hind-limb paralysis, followed by death. The virus was carried through another 3 rounds to obtain a stock which was titrated, aliquotted and stored in liquid nitrogen. Depending on virus titer, signs of sickness became evident as early as day 2-3 post-challenge. Based on a dose-response experiment using the virus stock prepared, we identified a dose (1.4×10^8^ pfu) that was adequate to cause 100% lethality within a week following i.p. administration (Figure S1), and used this dose for challenging DENV-E VLP-immunized mice to assess protective efficacy.

**References**

Arora D, Chauhan A, Khanna, N (1998) Easy PCR screening of *Pichia pastoris* transformants. Cell Mol Biol Lett 3: 21-24.

Batra G, Gurramkonda C, Nemani SK, Jain SK, Swaminathan S, Khanna N (2010) Optimization of conditions for secretion of dengue virus type 2 envelope domain III using *Pichia pastoris*. J Biosci Bioengg 110: 408-414.

Shresta S, Sharar KL, Prigozhin DM, Beatty PR, Harris E (2006) Murine model for dengue virus-induced lethal disease with increased vascular permeability. J Virol 80: 10208-10217.
